# Effect of Oral Skim Milk Administration on Skeletal Muscle Protein Synthesis after Total Gastrectomy in Rat

**DOI:** 10.3390/nu16152390

**Published:** 2024-07-23

**Authors:** Atsushi Sawada, Ryo Takagi, Junya Takegaki, Naoki Fukao, Koki Okumura, Satoshi Fujita

**Affiliations:** 1 Department of Physical Therapy, School of Rehabilitation Sciences, Health Sciences University of Hokkaido, Tobetsu 061-0293, Japan; as-51@hoku-iryo-u.ac.jp; 2 Graduate School of Sport and Health Science, Ritsumeikan University, Kusatsu 525-8577, Japan; 3 School of Nursing and Rehabilitation Sciences, Showa University, Yokohama 226-8555, Japan; r-takagi@nr.showa-u.ac.jp; 4 Graduate School of Agricultural Science, Kobe University, Kobe 657-8501, Japan; takegaki@whale.kobe-u.ac.jp; 5 Faculty of Sport and Health Science, Ritsumeikan University, Kusatsu 525-8577, Japan

**Keywords:** gastrectomy, skeletal muscle, protein synthesis

## Abstract

Leucine is a branched-chain amino acid that is present in protein, and it is an essential factor in activating the mechanistic target of the rapamycin complex 1 signaling pathway and increasing muscle protein synthesis. However, the loss of digestive function after total gastrectomy leads to impaired protein absorption, potentially failing to stimulate muscle protein synthesis. Therefore, this study aimed to investigate whether muscle protein synthesis is enhanced by oral skim milk administration after total gastrectomy. Male Sprague Dawley rats were divided into total gastrectomy (TG) and sham surgery (S) groups. After five weeks postoperatively, we orally administered skim milk to achieve 3.1 g protein/kg body weight and collected blood and gastrocnemius muscle. The gastrocnemius muscle weight was significantly lower in the TG group than in the S group (*p* < 0.05). The increase in plasma leucine concentration was significantly lower in the TG group than in the S group (*p* < 0.05). The skeletal muscle protein synthesis and the phosphorylation of p70S6K and 4E-BP1 showed a similar increase in both groups. Even after TG, muscle protein synthesis was stimulated by consuming skim milk, accompanied by a sufficient rise in plasma leucine concentration.

## 1. Introduction

In recent years, a decrease in weight and muscle mass has strongly impacted the overall survival of gastric cancer patients [1,2,3]; therefore, preventing the loss of weight and muscle mass is a significant challenge in gastric cancer treatment [4]. After total gastrectomy (TG), food administration will likely decrease due to the loss of gastric capacity [5]. Furthermore, several changes in protein digestion and absorption occur. Previous studies have revealed that the secretion of trypsin, a protein-degrading enzyme secreted from the pancreas, is reduced due to decreased pancreatic exocrine function resulting from diminished gastric secretion function [6,7]. Additionally, the loss of gastric capacity shortens small bowel transit time, leading to inadequate mixing of food and digestive enzymes [8] and abnormal bacterial overgrowth in the small intestine, decreasing protein absorption [9]. Consequently, addressing these nutritional disorders is crucial to preventing muscle mass reduction for post-gastrectomy patients. It is essential to elucidate nutritional support methods to avoid a reduction in muscle mass in patients after TG.

The muscle protein synthesis in regulating muscle mass is influenced by nutritional factors, particularly the administration of proteins and amino acids. A molecular mechanism within muscle cells implicated in promoting muscle protein synthesis is the mechanistic target of the rapamycin complex 1 (mTORC1) signaling pathway [10]. Leucine, a branched-chain amino acid (BCAA), is a nutritional factor involved in activating the mTORC1 signaling pathway. When leucine is taken up by muscle cells, mTORC1 is activated, leading to the phosphorylation of S6K1 (70-kD ribosomal protein S6 kinase) and 4E-BP1 (eukaryotic initiation factor 4E-binding protein 1), thereby promoting the synthesis of muscle proteins [11,12]. The amino acid concentration within muscle cells reflects the blood amino acid concentration [13]. Therefore, increasing leucine concentration in the blood is essential for initiating muscle protein synthesis.

The impact of gastric resection on regulating muscle protein metabolism has gradually been elucidated. Haba et al. reported no changes in the phosphorylation of S6K1 in the skeletal muscles at rest in rats that underwent TG [14]. Additionally, Wang et al. demonstrated that the intravenous administration of BCAAs to partial gastric resection rats stimulated muscle protein synthesis through the mTORC1 signaling pathway [15]. Therefore, increasing blood BCAA levels and activating the muscle protein synthesis pathway may be adequate as a nutritional strategy for mitigating skeletal muscle reduction after TG. The early initiation of oral administration after TG reduces postoperative complications and contributes to the early recovery of physical function [16]; therefore, it is widely employed as a standard treatment in many hospitals. However, the loss of gastrointestinal function after TG can lead to an impaired absorption of proteins [9]. Compared to an intravenous administration of amino acids, oral protein administration may not sufficiently elevate blood amino acid levels, potentially preventing the activation of the muscle protein synthesis pathway. Skim milk has been reported to be high in leucine [17] and cause a rapid increase in blood amino acid concentrations compared to other proteins [18]. However, previous studies have not conclusively demonstrated whether the oral administration of skim milk activates the muscle protein synthesis pathway after TG.

Therefore, this study aims to create a TG model in rats and verify the changes in blood amino acid concentration and the activation of the muscle protein synthesis pathway by administering oral skim milk administration. This study hypothesizes that activation of the muscle protein synthesis pathway after skim milk administration will be attenuated due to a reduction in blood amino acid concentration and subsequent mTOR signaling pathways in TG rats.

## 2. Materials and Methods

### 2.1. Experimental Animals

Seven-week-old male Sprague Dawley rats were obtained (Japan SLC, Inc., Hamamatsu, Japan). All rats were kept for two weeks under a 12-h light–dark cycle, and constant temperature and humidity (24 ± 1 °C, 50–60%). They were provided free access to water and standard solid feed (protein 23.6%, fat 5.3%, carbohydrates 54.4%, crude ash 6.1%, crude fiber 2.9%/MF, Oriental Yeast Co., Ltd., Tolyo, Japan). After two weeks of acclimation, the rats were divided into the total gastrectomy group (TG group, *n* = 5) and the sham surgery group (S group, *n* = 5). The rats were humanely sacrificed without distress under isoflurane anesthesia. All experimental procedures performed in this study were conducted according to institutional guidelines for the care and use of laboratory animals, as approved by the Animal Ethics and Research Committee of the Health Sciences University of Hokkaido (Approval number 059, approved on 17 May 2017).

### 2.2. Experimental Design

The TG rat model was created using the method described by Koizumi et al. [19]. Rats weighing 280–300 g at nine weeks old were operated on under isoflurane inhalation anesthesia. TG was performed through a median abdominal incision, preserving the vagus nerves. Reconstruction was performed using the Roux-en-Y reconstruction method. The jejunum was ligated and cut 2–3 cm from the Treitz ligament, and the esophagojejunostomy was performed on the proximal side. The jejunojejunostomy was performed 4–5 cm distal to the anastomosis. For the control group, rats underwent a median abdominal incision under isoflurane inhalation anesthesia, and the stomach and jejunum were exposed without further surgical procedures before closing the abdomen.

Postoperatively, the rats were prohibited from oral administration for the first 12 h, and from 12 to 24 h postoperatively, only water was allowed. From 24 h after surgery, the rats were provided with free access to a water nutrition gel (DietGel Recovery, ClearH_2_O). From 48 h postoperatively, the rats were allowed ad libitum access to water and standard solid feed. After surgery, the rats were housed for five weeks, and body weight and food administration were measured weekly. Food administration was calculated as the difference between the weekly and remaining feeding amounts.

Table 1 shows the nutritional value of the skim milk used in this study (Morinaga Milk Industry Co., Ltd., Tokyo, Japan). Skim milk, amounting to 3.1 g of protein/kg body weight (BW), was dissolved in 3.0 mL of distilled water. After an overnight fast, the rats were orally administered the entire mixture. Muscle samples from the gastrocnemius muscle and epididymal white adipose tissue were collected under isoflurane anesthesia 60 min after protein administration. All samplings were conducted between 1 and 6 h after the start of the light period. Blood samples were collected from the tail vein before and 60 min after protein administration. The collected blood was gently mixed with EDTA in the blood collection tube and promptly cooled on ice. After centrifugation (4 °C, 2100× *g*, 10 min), plasma was obtained. All samples were frozen in liquid nitrogen and stored at −80 °C until analysis.

### 2.3. Plasma Free Amino Acid Concentration

To determine the plasma amino acid concentrations, centrifugal separation (4 °C, 7000× *g*, 10 min) was performed with the supernatant plasma mixed with 15% sulfosalicylic acid. The supernatant was re-centrifuged (4 °C, 14,000× *g*, 30 min) using an ultrafiltration filter, and the lower layers were collected and taken as samples after protein removal [20,21]. Amino acid concentrations were analyzed using a high-speed analyzer (L-8900; Hitachi, Tokyo, Japan). Amino acids were separated using ion exchange chromatography and were detected spectrophotometrically after post-column reaction with ninhydrin. Forty types of amino acids and related molecules were measured, among which the following amino acids were used in the analysis: histidine, isoleucine, leucine, lysine, methionine, phenylalanine, threonine, tryptophan, and valine.

### 2.4. Western Blotting

Muscle samples were homogenized in a radioimmunoprecipitation buffer containing a complete Mini protease and PhosSTOP phosphatase inhibitor cocktail (Sigma-Aldrich, St. Louis, MO, USA). The homogenates were centrifuged at 10,000× *g* for 10 min at 4 °C. The samples were then diluted in sample buffer and boiled at 95 °C for 5 min. Ten micrograms of protein were separated by sodium dodecyl sulfate–polyacrylamide gel electrophoresis and transferred to the membranes. The membranes were blocked with powdered milk and incubated overnight at 4 °C with a primary antibody: puromycin (MABE343, Merck Millipore, Burlington, MA, USA), total-p70S6K (#2708, Cell Signaling Technology (CST), Danvers, MA, USA), phosphor-p70S6K (Thr389, #9234, CST), or total-4E-BP1 (#9644, CST), phosphor-4E-BP1 (Thr37/46, #9459, CST). The membranes were then incubated for 1 h at room temperature with the appropriate secondary antibodies. Chemiluminescent reagents were used for signal detection. Ponceau S staining was used to verify equal loading between lanes and normalization. Band intensities were quantified with Image J software version1.53a (National Institutes of Health, Bethesda, MD, USA).

### 2.5. Muscle Protein Synthesis Rate

Muscle protein synthesis was measured according to the in vivo surface sensing of translation (SUnSET) method by Goodman et al. [22]. At 30 min after protein administration, puromycin (Lot. PTR2713, Fujifilm Wako Pure Chemical Corporation, Osaka, Japan) dissolved in phosphate-buffered saline at 0.04 µmol/g BW was intraperitoneally injected into the rats. The gastrocnemius muscle was collected 30 min after puromycin injection. Following homogenization, as described above, and centrifugation at 2000× *g* for 3 min at 4 °C, the supernatant was collected and processed for Western blotting. Puromycin incorporation was detected by summing the intensities of all protein bands in the Western blot.

### 2.6. Statistical Analysis

Measured values are presented as mean ± standard error. Unpaired *t*-tests were used to compare both groups. Statistical analysis was performed using IBM SPSS Statistics (version 26), with a significance level set at 5% for all tests.

## 3. Results

Table 2 displays the changes in body weight, food administration, and tissue weights. The postoperative weight at five weeks was significantly lower in the TG group compared to the S group (*p* < 0.05). The average food administration over the five weeks postoperatively was similar in both groups (*p* = 0.27), and the food intakes in both groups slightly increased from their preoperative levels. Gastrocnemius weight was significantly lower in the TG group compared to the S group (*p* < 0.05). However, the gastrocnemius weight per body weight was similar between the two groups (*p* = 0.32). Furthermore, epididymal white adipose tissue weight was significantly lower in the TG group compared to the S group (*p* < 0.05). The epididymal white adipose tissue weight per body weight did not significantly differ between the two groups (*p* = 0.11).

**Figure 1:**
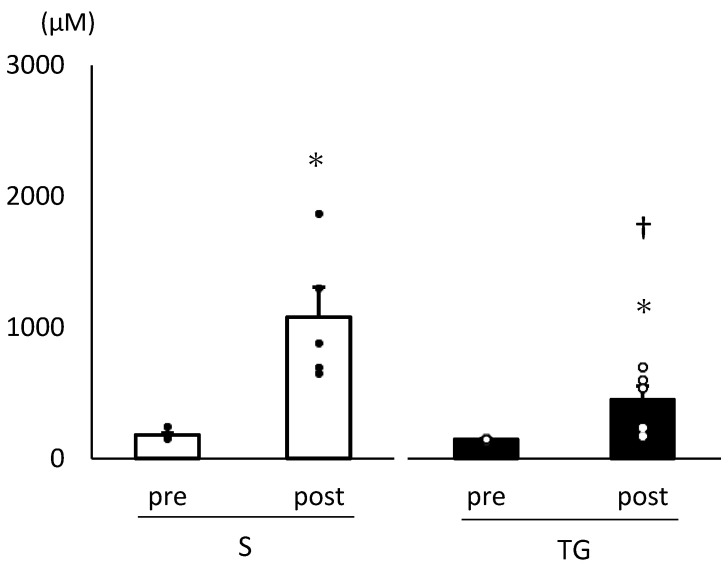
Plasma leucine concentration. We conducted a two-way analysis of variance and performed post hoc comparisons with a *t*-test. The results are presented as mean ± standard error. * Significantly different from the pre-values within the same group, *p* < 0.05. † Significantly different between groups, *p* < 0.05. pre, before protein administration; post, 60 min after skim milk administration; TG, total gastrectomy group; S, sham surgery group.

Figure 1 presents the changes in plasma leucine concentrations. Following skim milk administration, the plasma leucine concentrations significantly increased in both groups (TG group: *p* < 0.05, S group: *p* < 0.05). Furthermore, the plasma leucine concentration 60 min after skim milk administration was significantly lower in the TG group compared to the S group (*p* < 0.05). Table 3 displays the changes in plasma amino acid concentrations. The BCAA concentration and essential amino acid concentration at 60 min after skim milk administration were lower in the TG group than in the S group (BCAA: *p* < 0.05, essential amino acid: *p* < 0.05).

**Figure 2:**
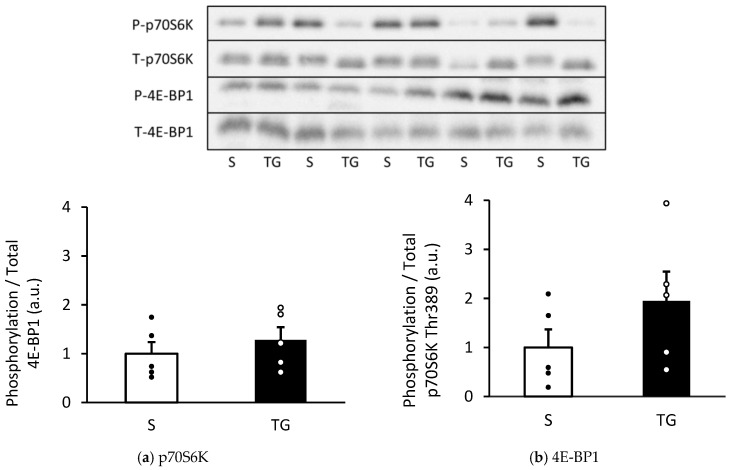
Changes in the phosphorylation of p70S6K and 4E-BP1 in the gastrocnemius muscle. Protein expression of phosphorylated p70S6K Thr389 (**a**) and4E-BP1 (**b**). The two groups were compared using an unpaired *t*-test. The results are presented as mean ± standard error. TG, total gastrectomy group; S, sham surgery group.

Figure 2 displays the result of the mTORC1 signaling pathways, which regulate the activation of protein synthesis. The phosphorylation of p70S6K and 4E-BP1 showed a similar level in both groups (p70S6K: *p* = 0.21, 4E-BP1: *p* = 0.45).

**Figure 3:**
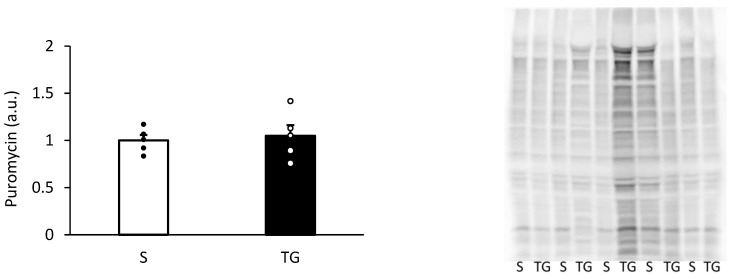
Muscle protein synthesis after the protein ingestion. The two groups were compared using an unpaired *t*-test. The results are presented as mean ± standard error. TG, total gastrectomy group; S, sham surgery group.

Figure 3 displays the result of muscle protein synthesis after the skim milk ingestion. Muscle protein synthesis was similar between the two groups (*p* = 0.71).

## 4. Discussion

We orally administered skim milk to rats with TG and investigated the fluctuations in plasma amino acid concentrations and the activation of the muscle protein synthesis pathway. We found that the plasma essential amino acid concentrations significantly increased 60 min after skim milk administration in the TG rats; however, the plasma amino acid concentrations at 60 min after skim milk administration were considerably lower than in the sham surgery rats. The activation of the mTORC1 signaling pathway after skim milk administration was similar between the groups. Additionally, muscle protein synthesis induced by skim milk administration showed a similar level in both groups. These results suggest that the increase in plasma amino acid concentrations after skim milk administration may be attenuated by TG. Nevertheless, in this study, the rise in plasma leucine concentration might have been sufficient to activate the muscle protein synthesis pathway. The result of this study demonstrates that even after TG, muscle protein synthesis would be stimulated by consuming skim milk accompanied by a sufficient increase in plasma essential amino concentrations. These findings provide insights for nutritional support to prevent skeletal muscle reduction after TG.

In this study, we administered 3.1 g/kg of protein in skim milk and measured plasma amino acid concentrations. This amount of protein corresponds to approximately 50% of the daily protein intake based on the dietary food intake over the five weeks post-surgery period in this study. Despite administering a large amount of protein, the plasma essential amino acid concentration in the TG group was significantly lower than the S group. The reason for this discrepancy remains to be investigated, but previous studies have reported that protein absorption transiently decreases due to small intestinal bacterial overgrowth after TG [9], suggesting changes in protein’s digestive and absorptive functions. Therefore, after TG, consuming a higher quantity of protein may be necessary to achieve sufficient elevation in plasma essential amino acid concentration. Additionally, the skim milk used was a high-quality protein with a high leucine content [17], and it was reported to increase plasma leucine concentration rapidly [18]. Therefore, proteins with a lower leucine content than skim milk, such as kidney beans [17], might not reach the plasma leucine concentration required to stimulate muscle protein synthesis. 

Oral administration of protein [23], BCAAs, and leucine [12] activates the mTORC1 signaling pathway in muscle protein synthesis. Elevating the concentration of BCAAs within muscle cells triggers this effect [24]. The intracellular BCAA concentration reflects the plasma BCAA concentration [13]. Consequently, elevating plasma BCAA concentrations, particularly plasma leucine concentrations, induces muscle protein synthesis [13,25]. We measured the phosphorylation of S6K1 and 4E-BP1 as markers of mTORC1 activity and simultaneously evaluated muscle protein synthesis. Despite the lower plasma leucine concentration in the TG group than the S group, the phosphorylation levels of S6K1 and 4E-BP1 were similar between the two groups. Additionally, muscle protein synthesis was also similar between the groups. Kanda et al. found that administering 1.54 g/kg body weight of milk protein to healthy rats significantly increased plasma leucine concentration to approximately 300 µmol, leading to an increased muscle protein synthesis after 60 min post-ingestion [23]. In this study, the TG group, which received 3.1 g/kg body weight of protein, showed an elevation in plasma leucine concentration up to 446 µmol. While this concentration was significantly lower than that of the S group, it was still sufficient to produce a similar activation of the mTORC1 signaling pathway and muscle protein synthesis. This implies that achieving a sufficient increase in plasma leucine concentration could ensure that the stimulation of muscle protein synthesis by leucine remains unaffected by TG.

In this study, the increase in body weight associated with growth was suppressed in post-gastrectomy rats. This result is similar to previous studies conducted in rats undergoing TG [14,19], indicating the validity of the TG model used. Despite maintaining food intake throughout the study, the growth of the TG group was restrained. This finding suggests the possibility of impaired digestion and the absorption of nutrients after TG. The plasma essential amino acid concentration 60 min after skim milk administration in the TG group was significantly lower than in the S group. This result may indicate changes in protein’s digestive and absorptive functions after TG. TG causes fat malabsorption, while carbohydrates are reported to be less affected [26,27]. Fat is hydrolyzed by pancreatic digestive enzymes, then micellized by bile acids, and subsequently absorbed through the intestinal mucosa [28]. After gastrectomy, the insufficient mixing of digestive enzymes and food occurs due to decreased pancreatic and bile secretion and the rapid emptying of food into the duodenum [26]. Furthermore, decreased gastric acid secretion leads to bacterial overgrowth in the small intestine, resulting in deconjugation of bile acids and impaired micellization of fats [27]. These factors collectively contribute to impaired fat digestion and absorption. In this study, the absolute weight of epididymal white adipose tissue was lower in the TG group compared to the S group. This finding suggests that impaired fat digestion and absorption due to total gastrectomy may inhibit weight gain in the TG group.

Preventing the loss of muscle mass in patients after TG is a significant challenge. Our study demonstrated that achieving a sufficient increase in plasma essential amino acid concentration including leucine can stimulate muscle protein synthesis, which may not be affected by TG. The elevation in plasma essential amino acid concentration induced by the intake of high-quality protein could potentially serve as a method to prevent a reduction in muscle mass in TG patients. However, after TG, it is recommended to increase meal frequency instead of reducing meal size per meal due to decreased gastric capacity [28]. Consequently, the use of supplements such as essential amino acids may be necessary to facilitate the increase in blood amino acid concentration and subsequent muscle protein synthesis. Further clinical research is necessary to determine the optimal nutritional support methods for TG patients.

The current study has several limitations. First, it did not evaluate the dose response when varying the skim milk administration. Consequently, this aspect needs to be clarified in this study. Second, it did not assess the temporal changes in blood amino acid concentrations in response to protein administration. Therefore, it cannot provide insights into changes in the digestive absorption function of proteins after TG. Third, the measurement of plasma glutamine or insulin was not conducted. A previous study has shown that in rat muscle after gastrointestinal surgery, the intracellular concentration of glutamine shows a positive correlation with muscle protein synthesis [2]. This suggests that the administration of skim milk may have affected muscle protein synthesis through changes in amino acid concentrations. In addition, the carbohydrates in skim milk may have stimulated muscle protein synthesis by increasing blood insulin levels [29]. However, studies have shown that combining carbohydrates with protein/amino acids does not produce a greater anabolic response than protein alone [30]. 

## 5. Conclusions

Skim milk was orally administered to rats with TG. As a result, while the increase in plasma essential amino acid concentrations in rats with TG was restrained compared to rats with sham surgery, the acute response of the muscle protein synthesis pathway was similar. These results suggest that even after TG, skeletal muscle synthesis may be stimulated by the adequate administration of high-quality protein. This study provides insights into nutritional support to prevent skeletal muscle reduction after TG. To tailor nutritional support, it will be necessary to investigate differences in protein dosage.

## Figures and Tables

**Table 1:** Composition of skim milk.

Nutritional Values (Composition Table, per 100 g)		
Calories	kcal	358.0
Protein	g	34.0
Fat	g	1.0
Carbohydrates	g	53.3
Salt equivalent	g	1.0
Calcium	mg	1200.0
Phosphorus	mg	960.0
Iron	mg	0.2
VitaminB1	mg	0.4
VitaminB2	mg	1.7

**Table 2:** Morphological change in experimental animals. Body weight and food administration are presented in (**a**), and gastrocnemius muscle and epididymis white adipose tissue are presented in (**b**).

**(a)**	**Body weight** **(g)**		**Food administration (g)**	
Group	Pre-surgery	5 weeks after surgery	Pre-surgery	Average for 5 weeks after surgery
TG	302.6 ± 5.2	286.9 ± 28.1 *	22.6 ± 0.5	23.9 ± 2.2
S	316.9 ± 8.1	449.5 ± 10.0 *	21.9 ± 0.5	25.7 ± 0.9
**(b)**	**Gastrocnemius muscle**		**Epididymis white adipose tissue**	
Group	Absolute weight	Weight per body weight	Absolute weight	Weight per body weight
	(mg)	(mg/g)	(mg)	(mg/g)
TG	1390.8 ± 167.4 *	5.2 ± 0.2	1289.7 ± 478.4 *	4.4 ± 1.3
S	2249.4 ± 70.1*	5.4 ± 0.1	2880.8 ± 156.5	7.0 ± 0.5

The two groups were compared using an unpaired *t*-test. The results are presented as mean ± standard error. * Significantly different between groups, *p* < 0.05. TG, total gastrectomy group; S, sham surgery group.

**Table 3:** Change in plasma amino acids concentrations.

		Pre	Post	Group × Time Effect	ES
BCAA (µM)	TG	441 ± 15.3	1110 ± 220.7 *	*p* = 0.02	0.49
	S	1357 ± 80.8	2652 ± 499.7 *†		
EAA (µM)	TG	1211 ± 45.2	2196 ± 329.0 *	*p* = 0.01	0.60
	S	1357 ± 80.8	5119 ± 773.3 *†		

We conducted a two-way analysis of variance and performed post hoc comparisons with a *t*-test. The results are presented as mean ± standard error. The effect size is presented as a partial η^2^. * Significantly different from the pre-values within the same group, *p* < 0.05. † Significantly different between groups, *p* < 0.05. Pre, before protein administration; post, 60 min after skim milk administration; ES, effect size; TG, total gastrectomy group; S, sham surgery group; BCAA, branched-chain amino acids; EAA, essential amino acids.

## Data Availability

Data are contained within the article.
